# Late Onset of ANCA Vasculitis as a Side Effect of Levamisole Treatment in Nephrotic Syndrome

**DOI:** 10.3390/medicina58050650

**Published:** 2022-05-11

**Authors:** Silvia Bernardi, Samantha Innocenti, Marina Charbit, Olivia Boyer

**Affiliations:** 1 School of Nephrology, Università degli Studi di Milano, ASST Papa Giovanni XXIII, 24127 Bergamo, Italy; silvia.bernardi1@unimi.it; 2 School of Nephrology, Università degli Studi di Firenze, AOU Meyer, 50139 Firenze, Italy; samantha.innocenti@unifi.it; 3 Néphrologie Pédiatrique, Centre de Référence du Syndrome Néphrotique Idiopathique de l’Enfant et de l’Adulte, Hôpital Necker-Enfants Malades, AP-HP, 75015 Paris, France; marina.charbit@aphp.fr; 4 INSERM U1163, Institut Imagine, Université Paris Cité, 75015 Paris, France

**Keywords:** ANCA-vasculitis, nephrotic syndrome, Levamisole, children, case report

## Abstract

Levamisole is effectively used in steroid-dependent nephrotic syndrome and the more frequent side effects reported are cytopenia and liver enzymes alterations. Several studies have demonstrated that this drug can induce high titers of circulating perinuclear antineutrophil cytoplasmic autoantibodies (ANCA) and vasculitis, most of them occurring in the case of prolonged use. A four-year-old boy that was affected with steroid-dependent nephrotic syndrome was treated with Levamisole as a steroid-sparing agent at a dose of 2 mg/kg/48 h. After initiation of the treatment, the number of relapses drastically decreased, enabling a significant reduction in the cumulative steroid dose. Levamisole was well tolerated, and was therefore administered for several years. At the age of 15, he was also diagnosed with celiac disease. After nine years of continuous Levamisole treatment, he presented with a high fever, hand and foot joint arthritis, and increased levels of total and direct bilirubin. Since the symptoms started two days after the injection of the second dose of the COVID-19 vaccine, it was initially concluded that these manifestations were rare vaccination side effects. Therefore, he did not receive any specific treatments, and Levamisole was continued at the same dose. After an initial improvement, two months later, the patient presented with the same symptoms. Suspecting Levamisole-induced vasculitis, an ANCA titer was measured and this returned positive. Clinical manifestations and double positivity for both myeloperoxidase (MPO) and anti-proteinase 3 (PR3) antibodies argued against the fact that that these findings were secondary to vaccination, cocaine abuse, or celiac disease. Assuming that we were facing a rare drug reaction, Levamisole was promptly interrupted. This resulted in a rapid remission of fever and arthritis improvement, and a decrease in ANCA titers. By reporting this case, we want to raise awareness among clinicians regarding a rare complication of treatment with Levamisole that is often misdiagnosed due to the fact that the current literature lacks univocal guidelines regarding the precise timing of ANCA titrations and the duration of the treatment.

## 1. Introduction

Idiopathic nephrotic syndrome is the most common glomerular disease in children, and presents with nephrotic range proteinuria, hypoalbuminemia, and decreased oncotic pressure leading to diffuse edemas or anasarca and dyslipidemia [1]. Nephrotic syndrome can lead to several major complications, such as severe infections and thromboses. Patients are classified according to their response to steroids. More than 90% achieve complete remission within 4 weeks of an oral course of steroids, and consequently are classified as being steroid-sensitive. Steroid-sensitive nephrotic syndrome evolves by relapses and remissions, and may persist in some patients into adulthood. Younger children tend to have relapsing courses [2]. Relapses are defined as 2+ proteinuria in a dipstick of first morning urine for at least three consecutive days, or 3+ proteinuria for at least two consecutive days. Around one half of children with steroid-sensitive nephrotic syndrome will relapse several times, requiring high cumulative doses of prednisone. Children may be steroid-dependent, if they experience two consecutive relapses while still on a steroid regimen, or within two weeks after complete withdrawal of steroids; or frequent relapsers, which are defined as having two or more relapses within six months of initial response, or more than four relapses in any 12-month period [3]. These conditions lead to prolonged exposure to steroids and possibly growth retardation, osteoporosis, adrenal suppression, cushingoid features, cataract, glaucoma, obesity, glucose intolerance, diabetes mellitus, bone demineralization, an increased risk of infections, arterial hypertension, and psychiatric disorders. Therefore, in the case of steroid-dependency or frequent relapses, steroid sparing agents are indicated as a second-line therapy. The most common of these are alkylating agents (cyclophosphamide), antimetabolites (mycophenolate mofetil), and calcineurin inhibitors (cyclosporine and tacrolimus). These drugs can also cause serious side effects. Levamisole, an imidazothiazole derivative used as an anthelminthic immunomodulator, is also effectively used in order to reduce cumulative steroid doses and the number of relapses [1,4,5]. It is generally well tolerated at a usual dose of 2–2.5 mg/kg/48 h. The main and most highly recognized side effects are neutropenia, mild anemia, gastrointestinal symptoms, and elevation of liver enzymes [6], while convulsions and ataxias are only scarcely reported [7,8]. Recent studies have demonstrated Levamisole-induced ANCA positivity and vasculitis, associated with a broad spectrum of clinical manifestations, mostly occurring in the case of prolonged use [9,10]. We present the case of a 15-year-old boy with a history of steroid-dependent nephrotic syndrome, treated with Levamisole as a steroid-sparing agent, who developed ANCA positivity and vasculitis, and we detail the diagnostic process that led us to interpret these findings as a Levamisole-induced reaction.

## 2. Case Presentation

A young boy of North African descent was diagnosed with idiopathic nephrotic syndrome in October 2010 at the age of four years, presenting with massive proteinuria and general edema. His family history was negative for kidney diseases. He was initially treated with oral prednisone at a daily dose of 60 mg/m^2^ for four weeks, and achieved complete remission within one week. Steroid therapy was progressively tapered off [4]. A few weeks later, he relapsed and rapidly developed steroid-dependency. From 2010 to 2012, this young boy presented with seven relapses, each being treated with high doses of prednisone. At the beginning of 2012, the patient was enrolled in a double-blind, randomized, controlled trial comparing the efficacy of Levamisole versus placebo, but he was randomized into the placebo group and experienced several relapses. Therapeutic options were then discussed with the parents and therefore, in June 2012, Levamisole was associated as a steroid-sparing agent at a dose of 2 mg/kg on alternate days (50 mg, three times a week). After initiation of the treatment, the number of relapses drastically decreased, enabling a significant reduction in the steroid doses. In June 2018, Levamisole dosage was adapted for his weight, and increased to a dose of 75 mg on alternate days, in association with a low dose of prednisone (5 mg on alternate days). This treatment was well tolerated, and it was successfully administered continuatively for nine years. It was decided that this treatment would be maintained during the pubertal growth peak to reduce the cumulative steroid dosage and to enable proper growth. The last relapse occurred in February 2018 during an upper respiratory tract infection, while the patient was receiving 12.5 mg of prednisone daily and 50 mg of Levamisole on alternate days. After increasing the dose of prednisone to 15 mg daily, the patient went into remission in 10 days. Blood counts were monitored every three months, and liver enzymes remained normal. Bone density measurements performed in 2021 did not reveal any alterations, and the only reported adverse effects of steroid therapy was mild acne.

In February 2021, at the age of 15 years, this boy complained of diarrhea, abdominal pain, and weight loss. In June 2021, the symptoms worsened and laboratory assessments showed a high degree of positivity for anti-transglutaminases (128 U/mL, normal values < 7 U/mL) and anti-endomysium antibodies (160, normal values < 10) associated with the HLA DQ2 genotype. IgA levels and thyroid function were normal, and anti-thyroglobulin antibodies were negative. A duodenal biopsy was then performed, confirming suspicions of celiac disease [11]. From July 2021, he then started a strict gluten-free diet, and achieved complete remission of the symptoms and a normalization of anti-transglutaminase antibodies within a few months.

The patient has had no documented history of COVID-19 disease, and in the second half of August 2021, he received the first dose of the Pfizer-BioNTech COVID-19 mRNA vaccine without complications. On 11 September 2021, he received the second dose. After two days, the patient developed a high fever, severe arthralgia of the hands and feet, and edema of the feet and ankles. Accidental trauma was not reported, and he was then referred to the Emergency Department. Vital signs were stable and a laboratory assessment noted elevated levels of reactive c-protein (38 mg/L, normal values < 5 mg/L), increased total bilirubin (28.10 µmol/L, normal values 5.1–20.5 µmol/L), and direct bilirubin (9.30 µmol/L, normal values 0–8.6 µmol/L) associated with normal levels of transaminase, gamma GT, alkaline phosphatase, and serum albumin. Kidney function and coagulation screening were normal, and only mild microcytic anemia was found (Hb 11.8 g/dL), without leukocytosis (Table 1). Considering the temporal relationship, diagnosis focused on an adverse reaction to the COVID-19 vaccine, and only careful monitoring was carried out, with progressive improvement of the symptoms in a few days. 

Unfortunately, at the beginning of December 2021, the patient complained of a new onset of severe joint pain affecting his hands and feet, peripheral edema, and a high fever. Joint mobility and flexibility were preserved. The first diagnostic hypothesis was a relapse of nephrotic syndrome, but urinary dipsticks were negative. Hemoptysis was not reported, and neither respiratory symptoms nor ocular or skin manifestations were noticed at clinical examination. Suspecting an adverse reaction to Levamisole, immunofluorescence tests were then performed for the first time, showing an elevated titer of ANA and ANCA, confirmed with both MPO (370 UI/mL, normal values < 20 UI/mL) and PR3 positivity (38 UI/mL, normal values < 20 UI/mL) when assessed using the ELISA method (Table 1). The blood count was normal and the treatment was then promptly interrupted. Furthermore, there is a reported correlation between cocaine and levamisole assumption and a new onset of ANCA vasculitis [12,13], and therefore drug abuse was also investigated, but a past history of cocaine or illicit drug consumption was negative. A few days after levamisole interruption, a complete resolution of symptoms was obtained. At last, after an evaluation in April 2022, the patient was in good clinical condition with low-dose prednisone (5 mg on alternate days), which is planned to be discontinued soon. Levamisole was not reintroduced, and no relapses occurred after its cessation. PR3 ANCA were negative, and MPO ANCA antibodies were persistently elevated, although their titers were slowly decreasing (Table 1). They will be further monitored, since persistently elevated levels have been reported up to several months after discontinuation [14,15].

## 3. Discussion

Patients affected with steroid-dependent nephrotic syndrome are frequently treated with steroid-sparing agents in order to reduce the side effects of the prolonged use of steroids. 

The treatment is still challenging, and there are no data from randomized controlled trials to determine which steroid-sparing agent should be used as the first agent [16,17]. In France, Levamisole may be used as a first-line therapy, especially in the case of frequently relapsing nephrotic syndrome or moderate steroid-dependency [4], and several studies have demonstrated its safety and efficacy in maintaining remission and in reducing steroid exposure [3,5]. However, in the case of prolonged use, especially after the second year of treatment, side effects are more frequent [3]. In September 2021, this patient developed systemic symptoms that were compatible with active vasculitis, including a high fever, fatigue, joints swelling, arthritis, and arthralgia associated with both MPO and PR3 ANCA antibodies. The kidney function and urinary sediment were normal. 

While ANCA vasculitis is a known side effect of Levamisole, it mostly manifests with cutaneous vasculitis, and more rarely, with systemic vasculitis. Kidney involvement, in particular, is scarcely reported in Levamisole-induced ANCA vasculitis. Similarly, the current literature reports that patients that are affected with ANCA-associated vasculitis that is not related to drug toxicity can present with a localized form, with only one organ involved, or an early systemic form, with no kidney involvement [18]. Although the diagnosis of ANCA vasculitis would have been better confirmed with a skin biopsy, this procedure is not systematically performed in clinical practice or trials, but the diagnosis rather is based on compatible clinical features and a positive ANCA serology only in the absence of kidney involvement [19].

Due to the fact that he had received the COVID-19 Pfizer-Biotech vaccine two days earlier, the diagnosis was focused on an adverse reaction to the vaccination, and therefore, Levamisole administration was continued. Common adverse events after the COVID-19 vaccine include mild reactions at the injection site, and fatigue, fever, and headache, but recent case reports have also described a temporal association between COVID-19 vaccination and *de novo* ANCA vasculitis [20]. However, the cases reported in the literature concern mostly adults, following the first injection. The patients presented more severe manifestations, including massive rhabdomyolysis, necrotizing vasculitis, and acute kidney injury, and required specific treatments such as intravenous steroids, Rituximab, and cyclophosphamide. Other authors described leukocytoclastic vasculitis after exposure to a COVID-19 vaccine, presenting with purpuric lesions and palpable papules, requiring antihistamines and prednisolone [21]. Our patient instead maintained a normal kidney function, creatinine kinase levels were in range, and clinical examination did not find suspicious skin manifestations; it was not necessary to administer specific treatments apart from Levamisole discontinuation. Moreover, in the published case reports, only MPO antibody titers were elevated, and the mean delay between vaccination and the first symptoms was 27 days [22,23,24]. Conversely, drug-induced vasculitis is associated with increased levels of both of these antibodies in the literature. Hence, the presence of both MPO and PR3 ANCA positivity in our patient argues against vaccine-induced ANCA vasculitis, but further supports Levamisole-induced ANCA vasculitis. Furthermore, the present patient experienced a second flare of the same symptoms, three months after, making the diagnosis of COVID-19 vaccine-induced ANCA vasculitis even less likely.

During the etiological diagnostic process, cocaine abuse was also investigated, since the literature has presented several cases of Levamisole detected in seized cocaine samples, mostly sold in United States, leading to Levamisole-induced vasculopathy and ANCA positivity. In fact, Levamisole contributes to a reduction in the cost of cocaine preparation, and it increases the effects and duration of this illicit drug [15]. Cocaine itself has been demonstrated to be associated with *de novo* ANCA-positive pseudo-vasculitis [12]. In cocaine users, rare cases of agranulocytosis have also been reported [13]. Urinary tests were not performed at the Emergency Department to exclude cocaine abuse. However, the child was oriented with no neurological symptoms, and normal cardiac frequency. Cocaine abuse, furthermore, is associated with other alterations such as hyponatremia, hypertension, rhabdomyolysis, renal infarction, and renal vein thrombosis [25], which our patient did not present. 

Analyzing the personal history, celiac disease was also considered to play a role, since ANCA antibodies may be found in inflammatory bowel diseases, microscopic forms of colitis, and in very rare cases of celiac disease. However, the symptoms of celiac disease began well before the symptoms of vasculitis—February 2021 vs. September 2021, with a second flare in December 2021—and disappeared rapidly with the introduction of the gluten-free diet, as did the relevant antibodies. This makes the concomitant occurrence of these two autoimmune diseases unlikely, even though this patient may be predisposed to autoimmune diseases, possibly due to his at-risk HLA allele. However, in those situations, immunofluorescence results are mostly positive for the perinuclear anti-neutrophil cytoplasmic antibodies (p-ANCA) pattern [26,27], and these findings are not usually associated with high fever and arthralgia. 

The presence of both MPO and PR3 ANCA positivity raised our suspicions for a drug culprit, and supported the hypothesis of a Levamisole-induced ANCA vasculitis [28]. It is also very interesting to note that the exams performed at the Emergency Department revealed, for the first time, increased levels of total and direct bilirubin. These values were initially interpreted as being a physiological response to high fever. However, hepatotoxicity has been described as a possible side effect of Levamisole, and these data support the thesis of a drug reaction, even if it is more frequently associated with elevated levels of transaminases [6]. After Levamisole discontinuation, general conditions promptly improved, and bilirubin levels returned to normal. A retrospective study performed in 2007 assessed both long- and short-term Levamisole effects in children affected with idiopathic steroid-dependent nephrotic syndrome, and demonstrated that after interruption, only a few children relapsed, suggesting that this drug may have a long-lasting effect [3]. Moreover, several studies have shown that elevated ANCA antibodies could also still be detectable after several months of Levamisole discontinuation [10], and therefore, we expect to find persistent ANCA positivity in the present patient within the next few months. 

## 4. Conclusions

Reporting this case, our aim is to raise awareness regarding this rare complication and illustrate that Levamisole-induced ANCA vasculitis may have a late onset, even after nine years of treatment. When symptoms suggestive for vasculitis are reported in patients treated with Levamisole, it is important to consider during the diagnostic process an adverse reaction, even after several years of treatment without complications. In these cases, ANCA antibody titration should be performed in order to promptly interrupt the therapy if necessary. After Levamisole discontinuation, nearly all patients have favorable outcomes [5] and ANCA titers progressively normalize. The current literature lacks univocal guidelines establishing the timing of ANCA monitoring while on therapy with this anthelminthic immunomodulator, leading to a huge degree of variability between centers and physicians. In fact, monitoring of ANCA titers is not recommended in the *Kidney Disease: Improving Global Outcomes* (*KDIGO*) documents published in 2012 or 2021 [16,17], nor in the *Summary of Product Characteristics*. It should be noticed that in the context of Levamisole toxicity, ANCA positivity may occur without specific clinical features, and Levamisole may have a long-term effect. Further studies are required to determine the appropriate duration of the therapy, and to define a safe upper limit of the dose and frequency of ANCA titration. Our aim is thus to encourage physicians to settle a precise timing of ANCA titration during Levamisole treatment, and to suggest that this rare but misleading adverse effect should not be underestimated.

## Figures and Tables

**Table 1:** Laboratory data.

Data	September 2021	December 2021	April 2022
Hemoglobin (g/dL)	13	11.6	13
Leucocytes (G/L)	5.51	3.91	5.83
Neutrophils (%, mm^3^)	35.7%, 1471 mm^3^	40.7%, 1591 mm^3^	45.7%, 2660 mm^3^
Eosinophils (%, mm^3^)	0.0%, 0 mm^3^	0.0%, 0 mm^3^	0.2%, 0.01 mm^3^
Basophils (%, mm^3^)	0.7%, 29 mm^3^	0.3%, 12 mm^3^	0.5%, 0.03 mm^3^
Lymphocytes (%, mm^3^)	50.5%, 2081 mm^3^	48.8%, 1908 mm^3^	43.7%, 2550 mm^3^
Monocytes (%, mm^3^)	13.1%, 540 mm^3^	10.2%, 399 mm^3^	9.9%, 580 mm^3^
Platelets (G/L)	261	246	206
Ferritin (ng/mL)	21		
CRP (mg/L)	38		
Bun (mmol/L)	2.10		
Serum creatinine (µmol)	69		
Na^+^ (mmol/L)	136		
K^+^ (mmol/L)	4.20		
Total serum protein (g/L)	72.7		
Albumin (g/L)	43		47
Urinary stick	Trace proteins	Trace proteins	Negative
Total bilirubin (µmol/L)	28.10		
Direct bilirubin (µmol)	9.30		
AST (UI/L)	26	17	
ALT (UI/L)	26	16	
GGT (UI/L)	15	13	
CPK (UI/L)	97		
ALP (UI/L)	164		
ANCA		1280	1280
MPO U/mL (N < 20)		370	200
PR3 U/mL (N < 20)		38	15

## Data Availability

Not applicable.
